# Evaluation of a clinical and translational research initiative: Developing and implementing a collaborative evaluation process in CAIRIBU

**DOI:** 10.1017/cts.2023.12

**Published:** 2023-02-08

**Authors:** Jennifer M. Allmaras, Kristina L. Penniston, Betsy Rolland

**Affiliations:** 1 Department of Urology, University of Wisconsin School of Medicine and Public Health, Madison, WI, USA; 2 Institute for Clinical and Translational Research and Carbone Cancer Center, School of Medicine and Public Health, University of Wisconsin-Madison, Madison, WI, USA

**Keywords:** Collaboration, evaluation, large research initiative, research network, translational research

## Abstract

Funding for large research initiatives, such as those funded through the National Institutes of Health U mechanism, has increased since 2010; however, there is little published research on how to evaluate the success of such initiatives. Here, we describe the collaborative evaluation planning process undertaken by the Interactions Core of the Collaborating for the Advancement of Interdisciplinary Research in Benign Urology (CAIRIBU) research community, a clinical and translational research initiative funded by the National Institute of Diabetes and Digestive and Kidney Diseases. Evaluation is necessary to measure the impact of our work and to allow for continuous improvement efforts of CAIRIBU activities and initiatives. We developed and implemented an iterative seven-step process that engaged the Interactions Core, NIDDK program staff, and grantees at each step of the planning process. Challenges faced in planning and implementing the evaluation plan included the time burden on investigators to submit new data for evaluations, finite time and resources for evaluation work, and the development of infrastructure for the evaluation plan. We call on funding agencies to include more explicit requirements for evaluation participation from grantees, as well as dedicated funding to support the evaluation process, in future funding opportunity announcements for large research consortia.

## Introduction

Scientific research is increasingly being conducted in networks in response to the understanding that groundbreaking, paradigm-shifting research requires the collaboration of investigators from across fields and institutions [1,2]. This collaboration is equally critical in Clinical and Translational Research, which requires integrating research across basic, clinical, and population sciences. National Institutes of Health (NIH)-funded cooperative agreement mechanism grants (U-mechanism grants) involve the coordination of several grants or resources and involve substantial involvement of NIH staff [3]. National Institute of Diabetes and Digestive and Kidney Diseases (NIDDK) funding for ‘other U’ grants (e.g., non-U01 mechanisms such as U34, UG3, UH3, UM1, and U24) as a percent of research project grant activity codes has increased from 2010 to 2020 [4]. However, there is very little published research on how to evaluate the success of such large research initiatives.

Evaluation is defined as the systematic collection and analysis of data related to the activities, characteristics, and results of a program in order to make judgements and recommendations to improve program processes and effectiveness and inform future program decisions [5]. This process of systematic data collection and analysis can be used to improve efforts related to research initiatives and can increase the potential to secure future funding to continue a cooperative agreement’s work by providing evidence of an initiative’s successes. Evaluation is also critical to the responsible stewardship of public funds.

This article describes the process for developing an evaluation plan of the NIDDK-funded initiative, Collaborating for the Advancement of Interdisciplinary Research in Benign Urology (CAIRIBU). CAIRIBU is an umbrella organization that brings together NIDDK-funded basic, clinical, and population science investigators devoted exclusively to investigating benign genitourinary (GU) diseases and disorders. Although the CAIRIBU initiative was formally formed in 2018, in 2020, NIDDK funded a U24 Interactions Core to help facilitate collaboration among the centers and programs that make up CAIRIBU. The aims of the Interactions Core are to provide scientific leadership within the nonmalignant urologic research space, foster collaboration, and outreach between CAIRIBU-affiliated investigators and those in the wider nonmalignant urologic research community, provide administrative support for events and initiatives that support the overall goals of CAIRIBU, and evaluate the efforts of the CAIRIBU initiative as well as the work of the U24 Interactions Core in effectively executing the above aims.

CAIRIBU does not choose or change the work proposed by the individually funded centers or programs that make up CAIRIBU, but rather, CAIRIBU facilitates and encourages interaction and collaboration among CAIRIBU-affiliated centers and programs. An underlying tenet of the CAIRIBU initiative is that the conjoined efforts of investigators from all points along the research continuum are required in order to understand the pathological mechanistic changes of the urogenital tract and establish clinically relevant models that may be used to test promising therapies. Therefore, in order to accomplish this, interdisciplinary and cross-disciplinary interactions that set the stage for effective research collaborations that can tackle the most critical questions related to the spectrum of nonmalignant GU diseases is required.

**Fig. 1.**
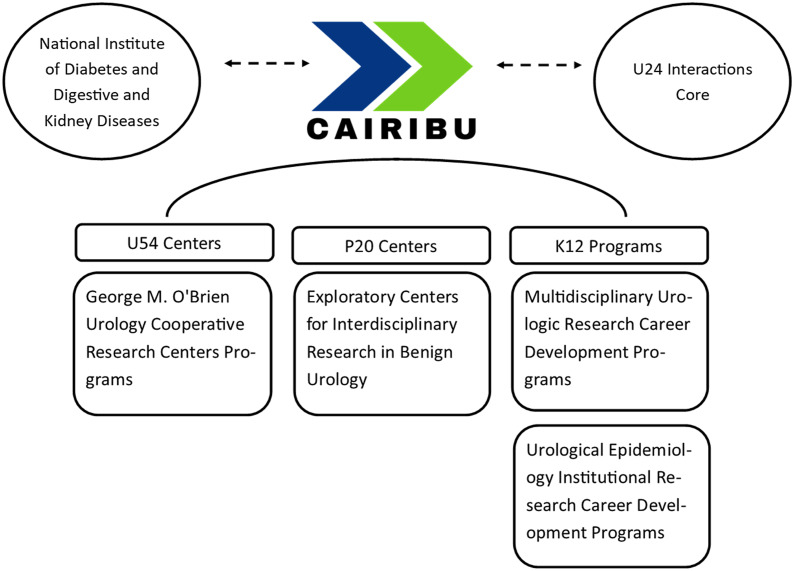
Centers and programs part of CAIRIBU. CAIRIBU, Collaborating for the Advancement of Interdisciplinary Research in Benign Urology.

The specific centers and programs vary from year-to-year as grant cycles end and others begin. In 2020, CAIRIBU included three types of participating centers and programs (Fig. 1). First, the largest centers within CAIRIBU are the George M. O’Brien Urology Cooperative Research Centers, U54-funded NIH/NIDDK grants. In 2020, CAIRIBU included three O’Brien Centers. Collectively, their research focused primarily on benign prostatic hyperplasia and congenital urinary tract malformations. Second, also in 2020, CAIRIBU included seven Exploratory Centers (P20) for Interdisciplinary Research in Benign Urology – 2-year center grants that were focused on bladder function, genomic and transcriptomic tools for studying the bladder and prostate, benign prostatic hyperplasia, lower urinary tract dysfunction/symptoms, kidney stones, nanotechnology applications in benign urology, machine learning for diagnosis risk stratification, and prediction of treatment responses in benign urological diseases, and the development of a urologic infection repository. Third, four K12 Career Development Programs were also part of CAIRIBU – 2 Multidisciplinary Urologic Research (KURe) Career Development Programs and 2 Urological Epidemiology (KUroEpi) Institutional Research Career Development Programs. These programs focus on training the next generation of benign urologic researchers.

Evaluation of the CAIRIBU initiative and the work of the U24 Interactions Core was written into the application for the U24 award. In that way, our team was able to incorporate an evaluative framework from the very beginning of Interactions Core’s work. This allowed our team to develop processes and methods for continuous reflection and improvement as the project progressed. It also allowed us to identify project successes, optimize these successes, and identify areas for improvement. Data collected through the evaluation process were also used to demonstrate the impact that CAIRIBU had on those within the consortium, thus encouraging participation and engagement in CAIRIBU initiatives. Findings from the evaluation established a baseline from which to understand change and helped us reach conclusions about the effectiveness of CAIRIBU efforts.

In order to achieve these goals, we followed a Collaboration Evaluation Planning Process designed by Dr. Betsy Rolland in her work with the National Cancer Institute’s (NCI) Healthcare Deliver Research Program (HDRP) on the evaluation of large research initiatives. The leadership of HDRP was interested in developing an approach to evaluation that would enhance their ability to assess the impact of their funded initiatives. Dr. Rolland conducted a comprehensive literature review of strategies for evaluating research initiatives and networks and a review of past NCI evaluations, along with short interviews with 19 program directors, and identified the most critical aspects of evaluation for large research initiatives. Working with the HDRP leadership, a process was developed that focused on *collaborative evaluation planning* that engages the NIH program staff, grantees, and the coordinating center or interactions core, where applicable. Here, we describe how that collaborative evaluation planning was implemented in CAIRIBU.

## Collaborative Evaluation Planning Process

**Fig. 2.**
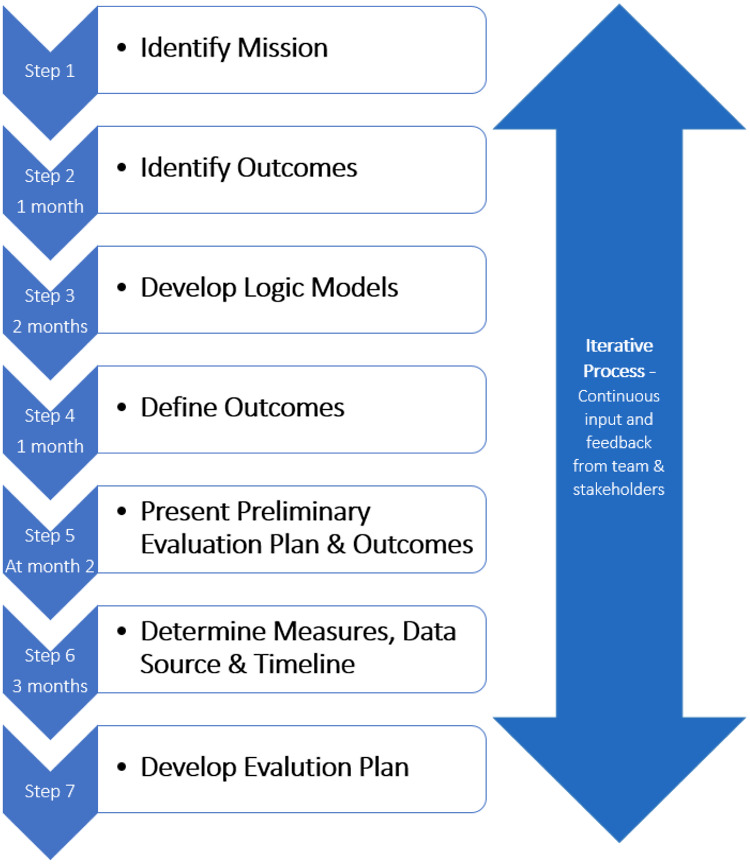
Seven-step iterative collaborative evaluation planning process.

The collaborative evaluation planning process of the CAIRIBU initiative involved seven steps. Figure 2 includes approximate time estimates for each iterative step. The team members involved in this process (4) met once a week for 1-hour team meetings; two team members met for an additional 30 minutes each week to work through the steps.

### Step 1: Identify Mission

Per the RFA (RFA-DK-19-034), the mission of CAIRIBU is to:

1. Support the next generation of urologic researchers by providing meaningful education, support, and mentoring
2. Cultivate new research tools and ideas by expanding the collaborative network within and outside of the traditional urologic research field
3. Enhance knowledge of mechanisms associated with normal development, function and disease pathology related to the urinary tract, kidney, and prostate
4. Translate the knowledge and tools generated from our collaborative work to the clinical setting to reduce the burden of benign urologic illness by developing and testing therapies to better treat, manage, and prevent these diseases

**Fig. 3.**
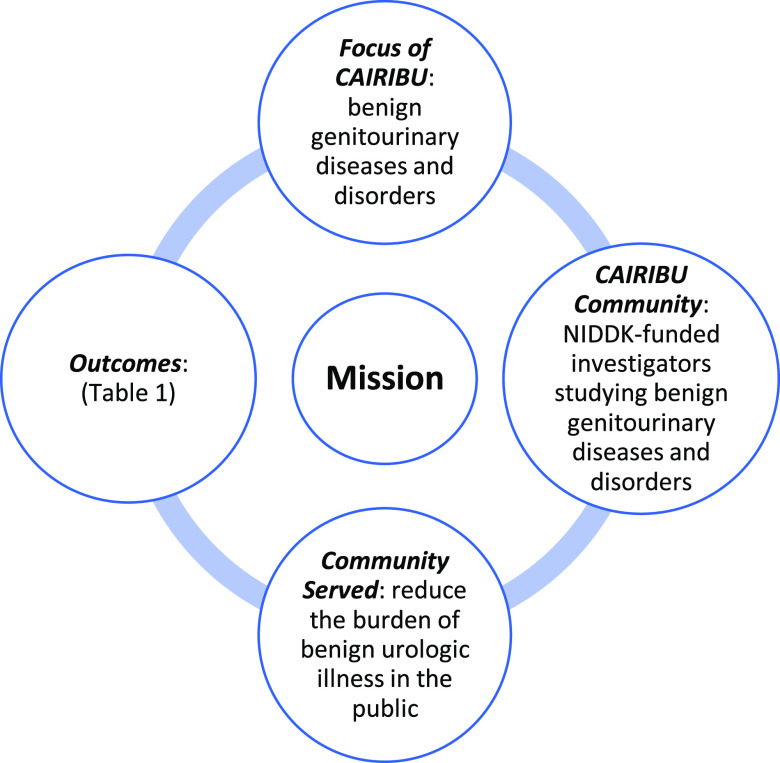
Dimensions of CAIRIBU’s mission. CAIRIBU, Collaborating for the Advancement of Interdisciplinary Research in Benign Urology; NIDDK, National Institute of Diabetes and Digestive and Kidney Diseases. Adapted from Gray Associates, Integrated Program Assessment and Management tool [12].

With this in mind, the first step in the collaborative evaluation process was to create an overarching evaluation framework that would inform priority setting. We then identified the core focus of CAIRIBU (Fig. 3) and the dimensions of CAIRIBU’s mission that were important to the evaluation plan (Fig. 3). These included identifying the specific communities involved and served and the specific outcomes that would demonstrate the CAIRIBU Community’s achievement of goals.

### Step 2: Identify Outcomes

The next step in the planning phase of the evaluation plan was to identify the outcomes that would indicate success of the CAIRIBU initiative and separate outcomes that would indicate success of the U24 Interactions Core. An outcome is an “event occurrence or condition that indicates progress toward achieving the purpose of the program” [6]. We defined the scope of the evaluation to include the CAIRIBU initiative, which formed in 2018, and the U24 Interactions Core, which began its work in 2020. Although CAIRIBU and the Interactions Core are interrelated, separate outcomes were identified because an outcome that might indicate success of the Interactions Core may not indicate success of the CAIRIBU Community as a whole. We defined outcomes related to CAIRIBU as those that involved interaction and collaboration between consortium centers and investigators while outcomes related to the Interactions Core were defined as those that involved the initiatives, services, and activities provided by the Interactions Core.

Outcomes for the evaluation plan were first identified from the initial U24 grant proposal and then through conversations with the Interactions Core team, which included the Interactions Core Principal Investigator (KP) and Co-Investigator (BR) and Interactions Core staff members (primarily JA). Challenges faced during this step included (1) determining the specific outcomes that both the CAIRIBU Community and the Interactions Core were working to achieve and (2) categorizing outcomes as either CAIRIBU cross-program interactions or Interactions Core activities and initiatives. After outcomes were initially identified, Interactions Core team members were asked to review them and to make any edits based on their knowledge of the overall goals of CAIRIBU and the Interactions Core. Next, the team was asked to mark the outcome as either a “CAIRIBU Community” or “Interactions Core” outcome. Feedback from the team was compiled and used to move forward with the next step of the planning phase. The time to complete this step was approximately 1 month. Outcomes are listed in Table 1.

**Table 1.** Measures, data source, and timeline for each outcome. Table divided by cross-program and interactions core outcomes

Outcomes and measures – cross-program interactions			
Outcome	Measures	Data source	Timeline
*Cross-program interactions outcomes*			
New investigators participate in CAIRIBU	Non-CAIRIBU investigators who participate in the annual meeting	Registration information	December
	Non-CAIRIBU investigators who participate as a grant reviewer	Reviewer spreadsheet	Collected after soliciting reviewers
	Non-CAIRIBU investigators who apply for CAIRIBU funding	Award tracking spreadsheet	Collected after each application closes
Trainees and early investigators participate in CAIRIBU leadership opportunities	Number of trainees who participate in leadership opportunities	Attendance tracking spreadsheet	Ongoing
CAIRIBU provides opportunities for higher-level trainees and early career faculty to understand interdisciplinary research and interact with investigators from other disciplines	Number of trainees who participate in Advancing the Advancing the Research Capacity of Trainees and Investigators at early-Career Stages (ARCTICS) community	Attendance tracking spreadsheet	Ongoing
	Trainee satisfaction with ARCTICS community	Survey to trainees after ARCTICS series	After final ARCTICS session
	Self-reported opportunities to initiate collaborative/mentoring relationships at ARCTICS events	Survey to trainees after ARCTICS series	After final ARCTICS session
	Description of ARCTICS	Planning in progress	Completed after sessions finalized
CAIRIBU provides opportunities for investigators to learn new things about the research and research tools used by other investigators	Changes in knowledge before and after scientific sessions (annual CAIRIBU meeting)	Post-CAIRIBU meeting evaluation survey	December
	Self-reported opportunities to learn new things about the research and research tools of other investigators	Post-CAIRIBU meeting evaluation survey	December
	Number of CAIRIBU events each year	Event tracking	Ongoing
CAIRIBU provides opportunities for people from diverse backgrounds to be part of the benign genitourinary field	Demographic information of annual meeting registrants	Registration information	Collected as investigators register for annual meeting
Increased engagement with cross-institutional research	Levels of Collaboration Survey	Levels of Collaboration Survey	Annually
	Self-reported interdisciplinary collaborations	CAIRIBU Progress Survey	Biannually
CAIRIBU investigators submit collaborative grant proposals	Number of new research teams created with CAIRIBU funding	Award tracking spreadsheet	Collected after each application closes
CAIRIBU investigators submit collaborative publications	Publication tracking of CAIRIBU community	PubMed	Ongoing
	Impact of CAIRIBU events on new collaboration	CAIRIBU Progress Survey	Biannually
	Assessment of Support for grant applications given within CAIRIBU	CAIRIBU Progress Survey	Biannually
The absolute number of successfully funded CAIRIBU investigators increases year over year	Grant tracking of CAIRIBU community	NIH RePORTER	Ongoing
*Interactions core outcomes*			
CAIRIBU-related events are featured in Interactions Core communications	Monthly website analytics of events page	Website Analytics Report	Ongoing
	Monthly newsletter open rate	Website Analytics Report	Ongoing
CAIRIBU maintains a presence at non-CAIRIBU events and meetings	Number of CAIRIBU-affiliated investigators who attend outside events	CAIRIBU at other meetings spreadsheet	Ongoing
	Inquiries about CAIRIBU from outside the community	Number of annual Contact CAIRIBU inquiries	Ongoing
Submissions for CAIRIBU grants and awards increase year over year	Number of investigators who apply for funds	Award Tracking Spreadsheet	Ongoing
Participation of the CAIRIBU community in opportunities to network and collaborate increases year over year	Participation at annual meeting and satisfaction	Attendance tracking spreadsheet; Post-meeting evaluation survey	December
	Participation in other CAIRIBU events/programs and satisfaction	Attendance tracking spreadsheet; Post-meeting evaluation surveys	Ongoing
	Participation in Center and Program events: symposia, etc.	Attendance tracking spreadsheet	Ongoing
CAIRIBU resources and training opportunities are accessed, including to create collaborative research proposals and publications	Website analytics of resources page	Website Analytics Report	Ongoing
	Website analytics of funding opportunities page	Website Analytics Report	Ongoing
	Website analytics of stakeholder engagement resources page	Website Analytics Report	Ongoing
	Assessment of utility of CAIRIBU resources and training opportunities to create collaborative research proposals	CAIRIBU Progress Survey	Biannually

CAIRIBU, Collaborating for the Advancement of Interdisciplinary Research in Benign Urology; ARCTICS, Advancing the Advancing the Research Capacity of Trainees and Investigators at early-Career Stages; NIH RePORTER, National Institutes of Health Research Portfolio Online Reporting Tools Expenditures and Results.

### Step 3: Develop Logic Models

**Fig. 4.**
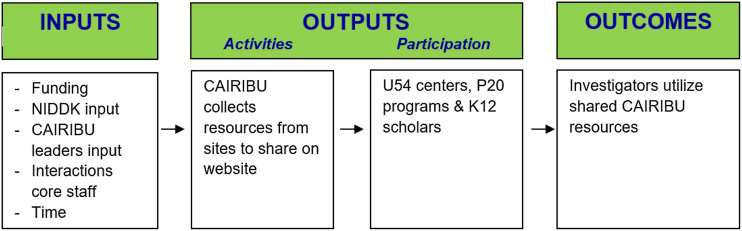
Snapshot of one outcome within the logic model. CAIRIBU, Collaborating for the Advancement of Interdisciplinary Research in Benign Urology; NIDDK, National Institute of Diabetes and Digestive and Kidney Diseases.

After preliminary outcomes were identified and categorized, two logic models were developed to show the relationship between program resources, activities, and outcomes using guidance from the “Developing a Logic Model: Teaching and Training Guide” [7]. Although logic models in the training guide are aimed at individual program evaluation, our team found it relatively straightforward to use the guide to develop a logic model for a large, multi-faceted scientific research initiative. Figure 4 shows a snapshot of the logic model for one outcome.

Logic models were presented to the team in order to provide a framework for how the goals of the program would be reached. As we progressed from this step, we returned periodically to update the logic models as needed over the course of 2 months (i.e. changes to outcome language, changes to logic model format, etc.).

### Step 4: Define Outcomes

The next step our team took was to have more in-depth conversations around our identified program outcomes. Outcomes were discussed one at a time to determine (1) the underlying goal of the outcome and (2) if the outcome was specific, measurable, achievable, realistic, and time-bound (SMART) [8]. The outcome language was altered, or reframed, to create more specific and measurable outcomes that gave the evaluation team clear direction on what data were needed to address each outcome. For example, an outcome that was originally phrased as “new investigators enter the field of benign GU research” became “new investigators participate in CAIRIBU.” We realized that the data needed to address the first outcome were murky and outside the scope of the Interactions Core whereas the data needed to address the second outcome were clear and within the scope of data we would be able to collect. This step helped our team create shared goals around the evaluation plan and a shared language for conceptualizing the outcomes. This step took approximately 1 month to complete.

### Step 5: Present Preliminary Evaluation Plan and Outcomes

Two months into the planning phase, a preliminary evaluation plan was presented to CAIRIBU Leaders, a group that includes Principal Investigators, Co-investigators, and Program Leaders/Managers of U54 George M. O’Brien Cooperative Research Centers for Benign Urology Research, P20 Exploratory Centers for Interdisciplinary Research in Benign Urology, and K12 Multidisciplinary Urologic Research and Urological Epidemiology Institutional Research Career Development Programs, and NIDDK Program Officers. By engaging these CAIRIBU leaders in the evaluation planning process and making adjustments based on their feedback, we ensured that the evaluation plan was reasonable, feasible, as low-burden (for CAIRIBU investigators) as possible, and capable of increasing CAIRIBU leader buy-in and ownership of the identified goals.

The presentation was led by the CAIRIBU Interactions Core Co-Investigator who shared information about the evaluation background and approach, the evaluation design process, collaborative evaluation planning, the evaluation scope, sample measures, and CAIRIBU outcomes. CAIRIBU Leaders were then placed in one of three virtual breakout rooms and given an outcome (finalized in step 4) to discuss. Discussions centered on defining the outcomes and listing possible measures to address the outcomes. Measures were compiled from each breakout room and used to inform step 6.

### Step 6: Determine Measures, Data Source, and Timeline

The next step was to determine the data to collect for each measure, the data source required, and the timeline for data collection. Outcomes and the associated measures identified in the previous step were listed on a spreadsheet along with measures suggested by the Interactions Core team members. A resource used in this step was the Partnerships for Environmental Public Health Evaluation Metrics Manual [9]. Several measures suggested by CAIRIBU leaders aligned with ideas our team had discussed previously. Such measures included inter-institutional collaboration as determined from PubMed data, tracking grants submitted and grants received, and use of CAIRIBU resources. The large majority of ideas suggested aligned with those suggested by our Interactions Core, which led us to conclude that the evaluation plan was progressing in such a way that supported CAIRIBU leader buy-in.

The Interactions Core team had weekly discussions to solidify the plan for data and data collection. Data to be collected fell into one of two categories: (1) pre-existing data accessible to the Interactions Core and (2) new data the Interactions Core would need to collect. Data from category one are data that are already being collected or can be sourced, for example, from NIH RePORTER, website analytics, attendance at events, and outcomes of various CAIRIBU pilot awards and other programs. Data from category two are those that need to be sourced from CAIRIBU investigators. As part of this process, two surveys were developed to collect the necessary metrics. These surveys were designed to be recurring at established time points throughout the year. To measure collaboration of centers and programs within the CAIRIBU Community, we modified the Levels of Collaboration Scale developed by Frey and colleagues [10]. The goal of this annual survey is to allow us to establish a baseline upon which to understand changes in collaboration between CAIRIBU centers and programs with time. To collect data on professional accomplishments, funding information, and collaboration at the individual levels, a second survey was developed to be sent to all active CAIRIBU investigators and trainees three times per year (CAIRIBU Progress Survey).

Due to the high burden associated with category two items, our team worked to maximize category one data as much as possible. Feedback and comments from the team were logged in a spreadsheet and used to create the final list of low burden, feasible measures, data sources, and a starting timeline. Going forward, these data will be stored securely on University of Wisconsin-Madison computers. A complete list of measures to be collected in the first 2 years of the funding cycle can be found in Table 1. The time to complete this step was approximately 3 months.

### Step 7: Develop Evaluation Plan

Lastly, a written evaluation plan was adapted from the Centers for Disease Control and Prevention’s Evaluation Plan Template [11] to include the following sections: (1) evaluation goal, (2) evaluation team, (3) stakeholder engagement, (4) background and logic models, (5) data collection and timeline, and (6) report and dissemination. The evaluation plan was presented to the Interactions Core team and feedback was incorporated to create the final evaluation plan to be presented to CAIRIBU Leaders for approval.

## Preliminary Evaluation Plan Implementation – Lessons Learned

**Fig. 5.**
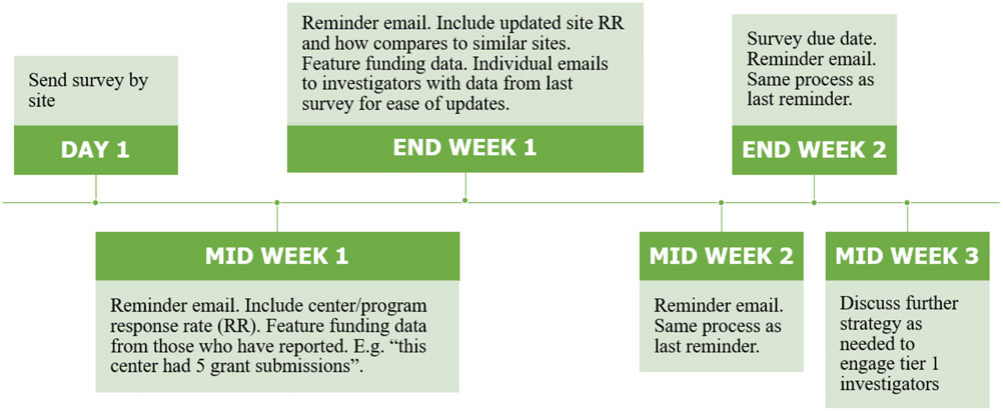
Standardized survey process for each survey part of evaluation plan. RR, response rate.

Data for the first step in implementing the evaluation plan were acquired through a pilot survey to all CAIRIBU investigators to collect data on their publications, collaborations with other sites, and submitted or funded grant proposals in order to create a baseline for measuring changes in collaborative activities. Our first experience fielding this pilot survey identified areas of process improvement, one of which was the development of a tier system to categorize investigators and trainees within the consortium into various response groups. For example, investigators from previously funded sites are encouraged to continue participating in CAIRBU-affiliated events and to continue to be known as ‘CAIRIBU investigators’; however, they are no longer high-priority for receiving evaluative communications such as surveys. Additionally, a survey process flow chart was developed to improve our response rate as well as to ensure that each time we sent out a survey or request for information, we followed the same process so as not to influence results by taking different steps each time (Fig. 5).

The development of a tier system and a standardized survey process increased our overall response rate for the same survey (CAIRIBU Progress Survey) from 25% (November 2021) to 47% (March 2022). After the second round of the survey, CAIRIBU leaders were asked again for feedback on the CAIRIBU Progress Survey to aid continuous improvement efforts. Leaders suggested sending out the survey at 6-month intervals rather than every 4 months to reduce investigator burden. Originally, it was thought that sending out the survey more frequently would be less burdensome as investigators would have to recall accomplishments during a shorter period; however, this was not the case. Instead, investigators suggested a process for providing their responses to earlier surveys at each 6-month interval to help them complete the subsequent survey more accurately.

Customized reports were developed using data collected through the initial survey and sent to leaders of each site prior to their Research Performance Progress Report (RPPR) due dates. Feedback on the reports was requested from site leaders through email and during monthly leadership meetings. Site leaders reported that the reports were useful but they did not offer any specifics for how they will use the data. Challenges around engaging leaders in the evaluation process remain. Soliciting feedback during monthly virtual leadership meetings has proven the most effective method for receiving feedback on CAIRIBU initiatives while feedback via email has proven the least effective method.

## Conclusion

Throughout the planning process, we frequently redirected our efforts, changed course, and restructured our process as we moved through the steps. Changes for best steps forward were based on new knowledge learned or feedback from our team or CAIRIBU leaders, including NIDDK Program Officers. We continually incorporated leader feedback into our process in creating the final CAIRIBU Evaluation Plan. The time burden on investigators to submit data for recurring evaluations remains a big challenge. Greater expectations of investigators around evaluation and its importance are needed. Specifically, we suggest a greater emphasis within funding announcements on evaluation – namely, the requirement to participate in evaluative efforts as a contingency of funding. Other limitations our team faced in developing the evaluation plan included finite time and resources as well as the hazards of implementing any new process. Therefore, it was essential to prioritize efforts related to data collection and analysis while also prioritizing feasibility without compromising the quality of the evaluation results. Preliminary evaluation results will inform continuous improvement efforts and provide guidance for developing and improving services and initiatives offered by the Interactions Core. Evaluation results will also provide baseline data for which to compare future data to improve conclusions about the results of CAIRIBU Community efforts.

While input on the evaluation of the CAIRIBU initiative was limited to NIDDK program staff and program grantees, the incorporation of community and stakeholder engagement at all levels within the CAIRIBU research community, including within the U24 Interactions Core, is a consideration for future efforts. In a 2020 paper, the NIDDK emphasized the importance of “engaging clinical trial participants more broadly in the research enterprise [to] advance scientific inquiry” [13]. Incorporating community and stakeholder engagement efforts in our evaluation process could result in the development of outcomes that reflect patient and/or other stakeholder priorities and also augment efforts to obtain more funding for nonmalignant urologic research. This might ultimately result in greater public attention on urologic diseases and conditions, more patient advocacy, a bigger urologic workforce pipeline, and in more scientific and therapeutic advances in the field.
